# Human herpesvirus 7 encephalitis in an immunocompetent adult and a literature review

**DOI:** 10.1186/s12985-022-01925-9

**Published:** 2022-11-29

**Authors:** Yi Li, Tianhao Qu, Dandan Li, Juanjuan Jing, Qiuming Deng, Xianyao Wan

**Affiliations:** 1 Department of Critical Care Medicine, The First Affiliated Hospital of Dalian Medical University, Dalian, 116021 China; 2 Department of Oncology, The First Affiliated Hospital of Dalian Medical University, Dalian, 116021 China

**Keywords:** HHV-7, Encephalitis, Immunocompetent adult, Epileptic seizures

## Abstract

**Background:**

Human herpesvirus 7 (HHV-7) is a common virus that infects children early and is accompanied by lifelong latency in cells, which is easy to reactivate in immunodeficient adults, but the underlying pathological mechanism is uncertain in immunocompetent adults without peculiar past medical history. Even though the clinical manifestation of the encephalitis caused by HHV-7 is uncommon in immunocompetent adults, the HHV-7 infection should not be neglected for encephalitis for unknown reasons.

**Case presentation:**

We reported here a case of HHV-7 encephalitis with epileptic seizures. While the brain computer tomography was standard, electroencephalography displayed slow waves in the temporal and bilateral frontal areas, then HHV-7 DNA was detected in the metagenomic next-generation sequencing of cerebrospinal fluid. Fortunately, the patient recovered after treatment and was discharged 2 months later. We also collected the related cases and explored a better way to illuminate the underlying mechanism.

**Conclusion:**

The case indicates clinicians should memorize HHV-7 as an unusual etiology of encephalitis to make an early diagnosis and therapy.

## Background

HHV-7 is a widespread double-strand DNA virus in the human population that belongs to the β-herpesvirinae subfamily and replicates in CD4^+^ T lymphocytes. The virus has the ability of lifelong latency and is easy to reactivate in immunocompromised patients [1, 2]. Primary HHV-7 infection regularly occurs during childhood and may manifest several clinical symptoms, mainly fever and exanthem subitum. As for reactivation of HHV-7, HHV-7 encephalitis is a severe type of infection with epileptic seizures as its clinical manifestation. However, there are uncertain aspects about the infection route, except for the history of the infectious agent in patients. Furthermore, on the one hand, as a lymphotropic virus, it has an obvious tropism for the central nervous system(CNS), but its neuropathogenesis is underestimated [3].On the other hand, the case of encephalitis associated with HHV-7 infection in an immunocompetent patient has been rarely reported.

Thus, we manifest an immunocompetent 26-year-old woman with encephalitis with HHV-7 infection and review current literature. With the treatment of acyclovir and ganciclovir, the patient recovered to normal.

## Case presentation

A 26-year-old female undergraduate presented to critical care medicine with a 29-day history of fever accompanied by mental and behavioral disorders. The body temperature rose, thus leading to a coma and intermittent convulsions during the last 28 days. Also, there was no significant personal history.

Before admission, she had received treatment in two hospitals. Her CSF sample was colorless, and routine analysis indicated total cells are 2/mm^3^, with 50% leukocytes, total protein and glucose levels were 0.341 g/L and 2.8 mmol/L, and CSF cultures were negative for bacterial and fungal organisms. Normal findings were obtained from the autoimmune encephalitis examination and cranial CT. EEG expressed irregular activity with epileptiform discharge in the anterior temporal and bilateral frontal areas. HHV-7 DNA was detected in the mNGS of CSF, she was diagnosed with meningitis and/or encephalitis in other hospitals, and then acyclovir was added. Sputum cultures were Acinetobacter baumannii and Burkholderia cepacia. After an active medical intervention, the body temperature was lower than the initial state, but progressive deterioration of the level of consciousness was uncontrolled and then led to a requirement for mechanical ventilation.

On admission, a physical examination revealed that she was unconscious and underwent mechanical ventilation via tracheostomy. Also, she had a fever, lung crackles, neck stiffness, and unconscious limb convulsions. A lumbar puncture was performed, and CSF pressure was 252 mm H_2_O. The patient was empirically treated with ganciclovir, meropenem, tigecycline, micafungin, corticoids, glycerol fructose, midazolam, levetiracetam, topiramate, perampanel, clonazepam, phenobarbital. On the third evolution day, mechanical ventilation mode was converted to oxygen inhalation mode, and her condition improved.

Furthermore, anti-ANA-IgG in serum was mildly positive, but a wide spectrum of antibodies (anti-nRNP, anti-Sm, anti-SS-A, anti-Ro-52, anti-SS-B, anti-Scl-70, anti-Jo-1, anti-ACA, anti-AnuA, anti-AHA, anti-ARPA, anti-AMA-M2, anti-PCNA, anti-PM-Scl100, anti-HHV-1-IgG, anti-HHV-2-IgG, anti-Toxo-IgM, anti-CMV-IgM, anti-Rubella-IgM) in serum were normal. To check the brain function, unfortunately, her cranial CT was negative, MRI was not performed because she had been wearing a dental appliance. The second mNGS of CSF indicated Enterococcus faecium and anti-Aspergillus-IgG in the blood were positive. Thus, we added contezolid and voriconazole to alleviate the previous treatment. On the 18th day of admission, her body temperature was up again, combined with primary epilepsy, lumbar puncture was performed, and CSF pressure was 210 mmH_2_O, but the CSF cultures and CSF mNGS were negative, then the blood antibodies (anti-HHV-1-IgG, anti-HHV-2-IgG, anti-Toxo-IgG, anti-CMV-IgG, anti-Rubella-IgG) was normal. With the treatment adjustment, the body temperature returned to normal, and the Glasgow Coma Score (GCS) was 15 points (E4, V5, M6). However, her memory ability was damaged, and she was transferred to a rehabilitation hospital for better treatment. Suddenly her body temperature rose again, accompanied by generalized epilepsy, then returned to our hospital the following day. The 4th CSF mNGS indicated HHV-1, EEG expressed irregular activity with a slow wave in the temporal and bilateral frontal areas, urine culture was Klebsiella pneumoniae, the patient was treated with contezolid, corticoids, levetiracetam, topiramate, Perampanel, Clonazepam, amika, ganciclovir. As time passed, her memory ability improved quickly, and she could eat by herself. Whereas she often felt choking when she ate chewy food, but the result of the gastroscopy indicated normal, to some extent implying she had a psychological disorder. Finally, with the help of humanistic concern and antiepileptic drugs, the patient recovered completely after two months.

The diagnostic evaluation of HHV-7 encephalitis needs to be guided by epidemiologic laboratory tests, imaging examinations, and clinical proof. The information is as follows. For the first line of investigation, HHV-7 DNA was detected in CSF, but there was no abnormal personal history (information provided by her relatives). For the second line of investigation, on day 1, the respiratory pathogen spectrum (Legionella, Mycoplasma pneumonia, Chlamydia pneumonia, Respiratory syncytial virus, Adenovirus, Influenza virus A, Parainfluenza virus, Coronavirus) were negative. However, Influenza-virus-B-IgM in serum was positive. Hepatitis viruses (Hepatitis A, Hepatitis B, Hepatitis C, Hepatitis E), Treponema pallidum, and Human immunodeficiency virus were negative, and sputum cultures were Acinetobacter baumannii and Burkholderia cepacia, we added antibiotics. On day 3, tubercle bacillus was negative, and bacterial cultures in the blood and CSF were negative. On day 7, a wide spectrum of blood antibodies (anti-nRNP, anti-Sm, anti-SS-A, anti-Ro-52, anti-SS-B, anti-Scl-70, anti-Jo-1, anti-ACA, anti-AnuA, anti-AHA, anti-ARPA, anti-AMA-M2, anti-PCNA, anti-PM-Scl100) were negative, anti-ANA-IgG was mildly positive, its titers were more than 1:100. However, these indications had no high specificity for the diagnostic of the disease. On day 8, Neisseria meningitides and Cryptococcal neoformans(serum and CSF) were also negative, then a wide spectrum of CSF antibodies (anti-HHV-1-IgG, anti-HHV-2-IgG, anti-Toxo-IgM, anti-CMV-IgM, anti-Rubella-IgM) was negative. Third line investigation, the cranial CT was negative, but the MRI was not performed because the dental appliance affected the result. Finally, on day 8, Enterococcus faecium was detected in the mNGS CSF, and we added contezolid combined with clinical proof. Then the mNGS of CSF indicated HHV-1 on day 33. The patient was treated with ganciclovir for about 6 weeks intermittently.

## Discussion

Viral encephalitis is the acute inflammatory phase of brain parenchyma induced by a viral infection. The clinical symptoms of it include fever and/or seizure; some patients with viral encephalitis cannot find the pathogenic virus. So the differential diagnosis of this patient excluded bacterial and fungal meningitis, malaria, structural brain lesions, toxic and metabolic encephalopathies, and other noninfectious causes of encephalopathy. The mNGS of CSF indicated HHV-7 DNA that supported HHV-7 as the most likely cause of encephalitis. After we added ganciclovir, the HHV-7 DNA was not detected. Due to the HHV-7 infection characterizations and the lack of HHV-7 avidity testing, we cannot distinguish between the secondary and the primary infection.

Among the viral encephalitides, HHV-7 encephalitis is associated with epilepsy. The seizures have been divided into late unprovoked and acute symptomatic. The current case represents the latter and may have a better outcome than the former [3]. Besides that, as for individual treatment, there is a huge gap between the expectation of antiviral therapy and the outcome of patients.

These cases in the table elucidate the similar clinical manifestation and the evolution of the cases in the literature. Patients’ ages ranged from 26 to 52, with a median of 35 years and only one female. The mortality was 14.2%, and ganciclovir was given to 6 patients [4–9]. Ganciclovir and foscarnet were recommended in immunocompromised patients with HHV-6 infection [10], but the treatment of encephalitis associated with the human herpesvirus-7 infection is still limited and unclear. It was common to see ganciclovir, acyclovir, foscarnet, and intravenous immunoglobulin therapy used in HHV-7 encephalitis. Also, some cases were improved without antiviral agents [11, 12]. In a retrospective analysis, we added ganciclovir empirically, but foscarnet had higher vitro activity than ganciclovir against HHV-7. The foscarnet was highly recommended in the literature [5, 9, 13]. Foscarnet would be a great choice for reducing the length of stay in the intensive care unit and the severity of HHV-7 encephalitis (Table 1).

**Table 1:** Review of similar cases with HHV-7 encephalitis in adults in the literature

Clinical diagnosis/immune status	Age	Gender	Primary infection/reactivation	Presentation	Cerebrospinal fluid		MRI/Brain CT	Antiviral therapy	Outcome		References
					PCR/mNGS	Other			Survival	Neurological deficit	
Encephalitis and inflammatory demyelinating disease/immunocompetent	28	Male	Primary infection	Headache,dizziness, fever, optic neuritis and epileptic-seizures	HHV-7 DNA in CSF (mNGS and PCR)	Anti-HHV-7 IgM and IgG in CSF,610 WBCs/mm^3^,protein 1.28 g/L, normal glucose	Hyperintensities involving the left frontal, orbital gyrus and bilateral optic nerve with substantial contrast enhancement (MRI)	Ganciclovir	Survived and remaining partially visually impaired	None	Li et al. [4]
Encephalitis/immunocompetent	44	Male	Reactivation	Gait impairment, anxiety, and urinary urgency	HHV-7 DNA in CSF (PCR)	60 lymphocytes/mm3,protein 0.449 g/L, normal glucose	Bilateral hyperintensities involving pyramidal tracts and basal ganglia with mild enhancement (MRI)	Ganciclovir、Foscarnet	Survived	Mild spasticity, hyperreflexia, and manageable sporadic urinary urgency	Corral et al. [5]
Encephalitis/immunocompetent	26	Male	NA	Fever, frontal headache, cervical and lumber pain, vomiting	HHV-7 DNA in CSF (PCR)	110 leucocytes, protein 0.82 g/L, glucose 0.6 g/L	Normal (MRI)	Acyclovir、Ganciclovir	Survived	None	Parra et al. [6]
Limbic encephalitis/immunocompetent	35	Male	Reactivation	General fatigue, fever, headache, vomiting, consciousness disturbance, and seizures	HHV-7 DNA in CSF (PCR)	Pleocytosis (35/mm3)with 94% lymphocytes, protein 0.765 g/L, glucose 1.13 g/L	High intensity areas in the bilateral hippocampi and periventricular white matter (MRI)	Acyclovir	Survived	None	Aburakawa et al. [7]
Neurosarcoidosis/immunocompetent	44	Male	Reactivation	Loss of visual acuity and papilledema	HHV-7 DNA in CSF (PCR)	Pleocytosis with 98% lymphocytes, protein 1.197 g/L, glucose 1.2 mmol/L	Papilledema(MRI)	None	Survived	NA	Martikainen et al. [8]
Encephalitis/stem cell recipient	52	Male	Reactivation	Impaired consciousness,short-term memory deficit,partial seizures	HHV-7 DNA in CSF(PCR)	Normal cell count and glucose and a mildly elevated protein level	Increased signal flair in the hippocampi(MRI)	Ganciclovir、Foscarnet	Died	NA	Holden [9]
Encephalitis/immunocompetent	26	Female	NA	Mental and behavior disorder, fever, coma, intermittent convulsions	HHV-7 DNA in CSF (mNGS)	1 leucocytes, protein 0.341 g/L, glucose 0.504 g/L	Normal (CT)	Acyclovir、Ganciclovir	Survived	None	Current case

*NA* not available; *PCR* polymerase chain reaction; *WBCs* white blood cells; *MRI* magnetic resonance imaging; *CT* computed tomography; *mNGS* metagenomic next generation sequencing

## Conclusion

As for unknown reasons of encephalitis cases present with seizures, the mNGS and PCR of CSF are options for identifying the potential causes, and HHV-7 infection could result in serious CNS disease that should be kept in mind. HHV-7 DNA has enough high specificity to guide the next treatment, and early clinical intervention is crucial, yet there is a great distance to search for the best treatment for HHV-7 encephalitis.

## Data Availability

Not applicable.
